# Prognostic value of the bone scan index using a computer-aided diagnosis system for bone scans in hormone-naive prostate cancer patients with bone metastases

**DOI:** 10.1186/s12885-016-2176-6

**Published:** 2016-02-19

**Authors:** Yasuhide Miyoshi, Shuko Yoneyama, Takashi Kawahara, Yusuke Hattori, Jun-ichi Teranishi, Keiichi Kondo, Masatoshi Moriyama, Shigeo Takebayashi, Yumiko Yokomizo, Masahiro Yao, Hiroji Uemura, Kazumi Noguchi

**Affiliations:** Department of Urology and Renal Transplantation, Yokohama City University Medical Center, 4-57 Urafune-cho, Minami-ku, Yokohama, 232-0024 Japan; Department of Urology, Yokohama Municipal Citizen’s Hospital, Yokohama, Japan; Department of Radiology, Yokohama City University Medical Center, Yokohama, Japan; Department of Urology, Yokohama City University Graduate School of Medicine, Yokohama, Japan

**Keywords:** Prostate cancer, BONENAVI, Bone scan index, Prognostic value, Hormone-naive

## Abstract

**Background:**

The bone scan index (BSI) using a computer-aided diagnosis system for bone scans is expected to be an objective and quantitative clinical tool for evaluating bone metastatic prostate cancer. This study aimed to evaluate the pretreatment BSI as a prognostic factor in hormone-naive prostate cancer patients with bone metastases.

**Methods:**

The study included 60 patients with hormone-naive, bone metastatic prostate cancer that was initially treated with combined androgen blockade therapy. The BONENAVI system was used for calculating the BSI. We evaluated the correlation between overall survival (OS) and pretreatment clinicopathological characteristics, including patients’ age, initial prostate-specific antigen (PSA) value, Gleason scores, clinical TNM stage, and the BSI. Cox proportional hazards regression models were used for statistical analysis.

**Results:**

The median follow-up duration was 21.4 months. Clinical or PSA progression occurred in 37 (61.7 %) patients and 18 (30.0 %) received docetaxel. Death occurred in 16 (26.7 %) patients. Of these deaths, 15 (25.0 %) were due to prostate cancer. The median OS was not reached. In multivariate analysis, age and the BSI were independent prognostic factors for OS. We evaluated the discriminatory ability of our models, including or excluding BSI by quantifying the C-index. The BSI improved the C-index from 0.751 to 0.801 for OS. Median OS was not reached in patients with a BSI ≤1.9 and median OS was 34.8 months in patients with a BSI >1.9 (*p* = 0.039).

**Conclusions:**

The pretreatment BSI and patients’ age are independent prognostic factors for patients with hormone-naive, bone metastatic prostate cancer.

## Background

Prostate cancer is the most common noncutaneous cancer, and the second most frequent cause of death from cancer among men in the USA. In Japan, 11,507 men were estimated to die of prostate cancer in 2014, making this disease the sixth leading cause of death from cancer [1]. The incidence of prostate cancer is lower in Japan than in the USA and other Western countries. However, this incidence has been gradually increasing in Japan in recent years [1]. Huggins and Hodges [2] reported the efficacy of androgen deprivation therapy for advanced prostate cancer in 1941. Although 80–90 % of prostate cancers with metastasis respond to initial androgen ablation therapy, most patients finally develop castration-resistant prostate cancer (CRPC) [3, 4]. Patients with CRPC show progression of systemic symptoms and local complications. One report showed that the median survival time among patients with advanced prostate cancer was 29 to 34 months from initial treatment [5], and another study reported a 5-year survival rate of 20–30 % [6]. Because these reports showed a wide range of survival probability, more accurate information on patients’ characteristics related to survival is required.

Several groups have reported prognostic models for survival of patients with progressive disease [7–10]. Most of these reports were of prognostic models for patients with CRPC. There are few reports on prognostic models for patients with metastatic prostate cancer before treatment. A large study on the prognosis of patients with pre-hormonal therapy prostate cancer was reported in Japan and the USA [9]. However, in this previous study, the endpoint was not survival, but recurrence. Previously, we reported a nomogram for overall survival (OS) of patients with bone-metastatic, hormone-naive prostate cancer [7]. This nomogram comprised five pretreatment prognostic factors (patient’s age, clinical T stage, classification of bone metastasis extension [extent of disease on bone scan, EOD scores] [11], Gleason scores, and PSA) selected by multivariate analysis. Among these prognostic factors, only EOD scores are subjective and semi-quantitative. Therefore, an objective and quantitative scoring system for evaluation of bone metastasis might be ideal and warranted. Recently, a computer-aided diagnosis system (BONENAVI) for bone scans has been developed. The BONENAVI system can calculate the “bone scan index (BSI)”, which provides an objective and quantitative measure of the percentage of the skeleton involved by bone metastases [12]. It is anticipated that a BSI that uses a computer-aided diagnosis system for bone scans will become an objective and quantitative clinical tool for evaluating bone metastatic prostate cancer. The BSI has been reported as being useful as a survival predictor among men with prostate cancer with various conditions such as hormone-naive prostate cancer or CRPC [13, 14].

In this study, we analyzed the relationship between the prognosis of prostate cancer and pretreatment clinical factors, including the BSI as calculated by BONENAVI for OS of patients with bone metastasis. This study aimed to determine whether the BSI is useful as a prognostic marker of hormone-naive, pretreatment prostate cancer with bone metastasis.

## Methods

### Patients and treatments

From 2010–2014, 60 consecutive patients with bone-metastatic prostate cancer were treated at Yokohama City University Hospital and associated hospitals. All of the patients already had metastasis at the time of diagnosis, and none of the patients had been previously treated. This study was a retrospective cohort study. This study was approved by institutional review board of Yokohama City University Medical Center and all subjects signed a written informed consent form.

All of the patients had adenocarcinoma of the prostate, confirmed histologically, with distant metastasis (any T, any N, M1b-c). The 2009 TNM clinical staging system and 2005 International Society of Urologic Pathology Gleason grading system were used. In all of the patients, the clinical stage was evaluated by chest and body computed tomography and bone scans. Based on the number or extent of metastases, the scans were divided into the following five grades according to the EOD on the bone scan [8]: 0, normal or abnormal due to benign bone disease; 1, fewer than six bony metastases, each of which is less than 50 % of the size of a vertebral body (one lesion approximately the size of a vertebral body was counted as two lesions); 2, from 6–20 bone metastases, sized as described above; 3, more than 20 metastases, but fewer than those observed in a “superscan”; and 4, a “superscan” or its equivalent (i.e., more than 75 % of the ribs, vertebrae, and pelvic bones).

Each hospital used the same treatment protocol. All of the patients were initially treated with androgen deprivation therapy (medical or surgical castration with or without anti-androgen). None of the patients received prostatectomies or radiation therapy as initial treatments. After failed initial androgen ablation therapy, almost all of the patients subsequently underwent substitution treatment comprising anti-androgen therapy, anti-androgen withdrawal therapy, and/or oral low-dose steroid therapy. Some patients received bisphosphonate and cytotoxic therapy, such as docetaxel or estramustine, after development of CRPC. None of the patients received abiraterone, enzalutamide, sipuleucel-T, or cabazitaxel because abiraterone, enzalutamide, and cabazitaxel were not approved until 2013, and sipuleucel-T has not been approved yet in Japan. In the terminal state, palliative therapy and pain control with morphine, palliative external beam radiation, and strontium were used as appropriate. Progression was defined according to recommendations of the Cancer Clinical Trials Working Group 2 (PCWG2) [15].

### Bone Scan Index (BSI)

The automated method for analysis of anterior and posterior whole-body bone scan images has been described previously [16]. Each individual hot spot was classified as metastasis or no metastasis by an artificial neural network. The BSI was calculated as the percentage of the sum of all hot spots classified as bone metastases by the artificial neural network values. For calculation of the BSI, we used BONENAVI version 2 (Fujifilm RI Pharma, Co. Ltd., Tokyo, Japan; Exini Bone, Exini Diagnostics, Lund, Sweden) [17].

### Statistical analysis

Spearman’s rank correlation coefficient was used for analysis of the correlation between EOD scores and the BSI. If there was a strong correlation, these two factors were not analyzed simultaneously because of multicollinearity.

We investigated the usefulness of a BSI as a predictor of OS. Univariate and multivariate analysis of OS was performed using a Cox proportional hazards regression model with stepwise regression analysis. Relative risks and 95 % confidence intervals (CIs) were derived. In univariate or multivariate analysis, all variables (age, PSA, Gleason scores, clinical TNM stage, and the BSI) were analyzed as binary variables. The cutoff point for all variables were determined using the median value of each variable. The C-index was used for discriminatory ability of our models.

The Kaplan–Meier product-limit estimator was used to estimate the distribution of survival. The log-rank test was used for analysis of the survival differences between patients with a BSI over the cut-off value and patients with a BSI under the cut-off value.

All tests were two-sided, and the significance level was fixed at alpha = 0.05. All analyses were conducted with IBM SPSS Statistics for Windows, ver. 22 (IBM Corp., Armonk, NY) and the R stats package (R Foundation for Statistical Computing, Vienna, Austria).

## Results

The characteristics of the patients are listed in Table 1. The median age of the patients was 72 years and the median follow-up duration was 21.4 months. The median initial PSA value was 247.0 ng/ml. The median BSI was 1.9 % (range: 0.0–13.2 %). Zoledronic acid was used for 28 (46.7 %) patients. Clinical or PSA progression occurred in 37 (61.7 %) patients and 18 (30.0 %) received docetaxel.

**Table 1:** Patients’ characteristics (*n* = 54)

Median age, years (range)	72 (55–89)
Median observation period, months (range)	21.4 (0.9–57.8)
Median PSA, ng/mL (range)	247.0 (9.7–4206.0)
PSA distribution, *n* (%)	
<10 ng/mL	1 (1.7)
10- < 20 ng/mL	2 (3.3)
20- < 100 ng/mL	15 (25.0)
100- < 500 ng/mL	20 (33.3)
≥500 ng/mL	22 (36.7)
Gleason scores, n (%)	
≤6	0 (0.0)
7	7 (11.7)
8	24 (40.0)
9	19 (31.7)
10	10 (16.7)
T stage, *n* (%)	
T1	0 (0.0)
T2	1 (1.7)
T3	43 (71.7)
T4	16 (26.7)
N stage, n (%)	
N0	24 (40.0)
N1	36 (60.0)
M stage, n (%)	
M1b	51 (85.0)
M1c	9 (15.0)
Median BSI, % (range)	1.9 (0.0–13.2)
EOD, n (%)	
1	21 (35.0)
2	20 (33.3)
3	15 (25.0)
4	4 (6.7)

*PSA* prostate-specific antigen, *BSI* bone scan index, *EOD* extent of disease

**Fig. 1:**
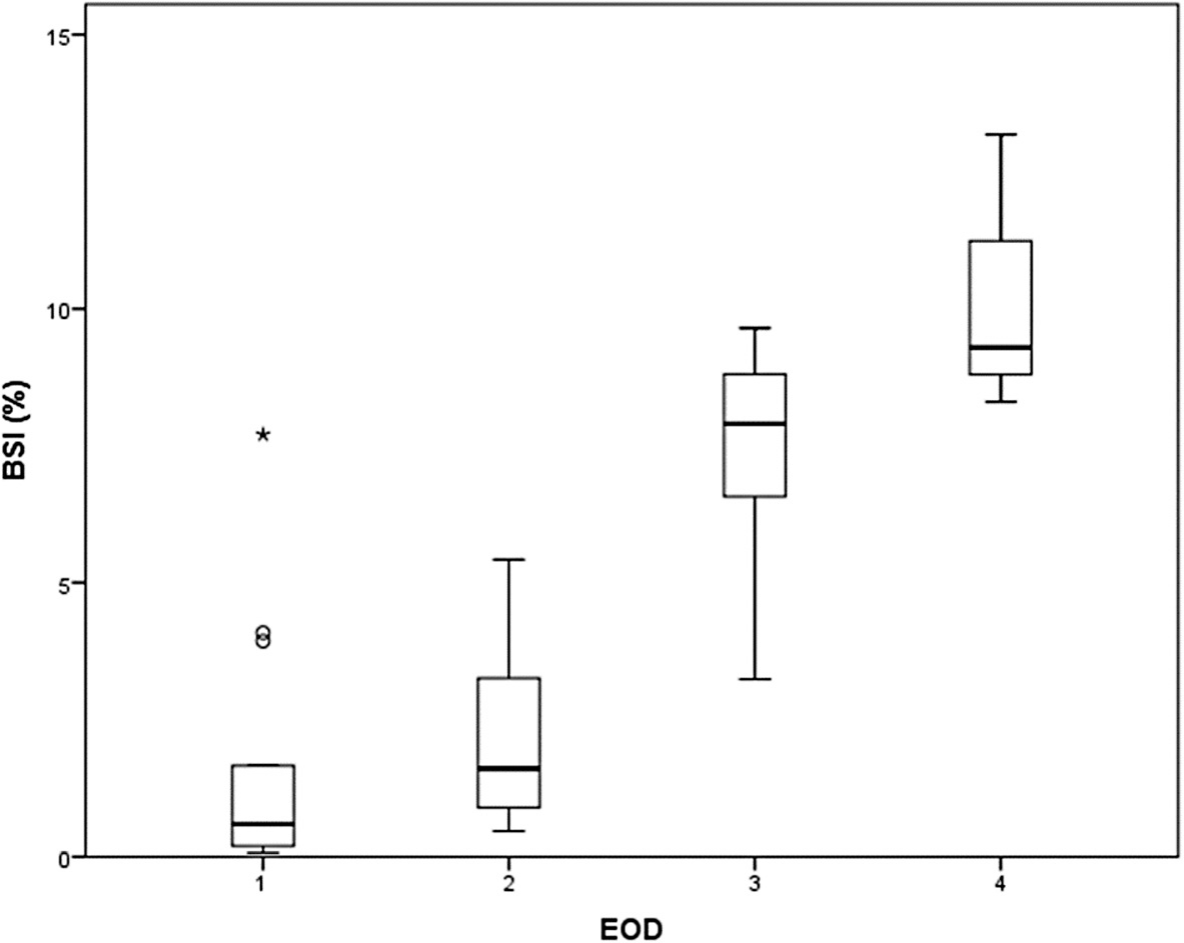
Correlation of EOD classification and the BSI. Box plots indicate the first and third quartiles. The band inside the box shows the median. Lines extending vertically from the boxes (whiskers) indicate variability outside the upper and lower quartiles. There was a significant correlation between EOD and the BSI (*rs* = 0.769). BSI; bone scan index, EOD; extent of disease on bone scan

The correlation of EOD scores and the BSI is shown in Fig. 1. The median BSI stratified by EOD 1, 2, 3, and 4 was 0.38, 1.60, 7.38, and 9.29, respectively. There was a significant correlation between EOD and the BSI (*rs* = 0.769). Because of the strong correlation, we did not analyze the BSI and EOD in multivariate analysis simultaneously owing to multicollinearity.

Death occurred in 16 (26.7 %) patients. Of these patients, 15 (25.0 %) deaths were due to prostate cancer. The median OS was not reached.

In univariate analysis, age (≤72 years old vs >72 years old, hazard ratio (HR): 4.136, 95 % confidential interval (CI): 1.332–12.843, *p* = 0.014) and BSI (>1.9 % vs ≤1.9 %, HR: 2.921, 95 % CI: 1.006–8.483, *p* = 0.049) were prognostic factors for OS (Table 2).

**Table 2:** Univariate and multivariate analysis for overall survival

	Univariate analysis				Multivariate analysis			
	*p* value	Hazard ratio	95.0 % CI		*p* value	Hazard ratio	95.0 % CI	
			Lower	Upper			Lower	Upper
Age (≤72 years old vs >72)	0.014	4.136	1.332	12.843	0.006	5.811	1.656	20.386
PSA (>247 ng/mL vs ≤247 ng/mL)	0.981	1.012	0.379	2.703	0.407	0.621	0.201	1.914
Gleason score (10–8 vs ≤7)	0.983	1.017	0.228	4.543	0.617	1.640	0.236	11.403
T stage (4 vs ≤3)	0.239	1.908	0.651	5.588	0.137	2.576	0.741	8.953
N stage (1 vs 0)	0.723	1.198	0.441	3.255	0.251	2.035	0.605	6.843
M stage (1c vs 1b)	0.744	0.780	0.176	3.462	0.467	0.524	0.092	2.990
BSI (>1.9 % vs ≤1.9 %)	0.049	2.921	1.006	8.483	0.023	4.676	1.238	17.661

*PSA* prostate-specific antigen, *BSI* bone scan index

In multivariate analysis, age (≤72 years old vs >72 years old, HR: 5.811, 95 % CI: 1.656–20.386, *p* = 0.006) and BSI (>1.9 % vs ≤1.9 %, HR: 4.676, 95 % CI: 1.238–17.661, *p* = 0.023) were independent prognostic factors for OS. (Table 2).

We evaluated the discriminatory ability of our models by quantifying the C-index. The C-index of our model including BSI was 0.801 for OS. We also analyzed the discriminatory ability of the model excluding BSI and the model including EOD instead of BSI by quantifying the C-index. The C-index of the models excluding BSI was 0.751 for OS. The C-index of the models including EOD instead of BSI was 0.811.

**Fig. 2:**
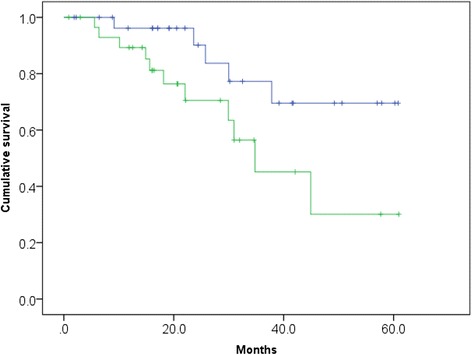
Kaplan–Meier curve for overall survival according to the bone scan index (BSI). The blue line indicates survival for patients with a BSI >1.9 (*n* = 30) and the green line indicates survival for patients with a BSI ≤1.9 (*n* = 30) (*p* =0.039)

We compared the survival probability by BSI categories. Median OS was not reached in patients with a BSI ≤1.9 and median OS was 34.8 months in patients with a BSI >1.9 (*p* = 0.039) (Fig. 2).

## Discussion

Several groups have reported prognostic models for survival of patients with progressive prostate cancer [7–10]. Almost all reports were of prognostic models of patients with CRPC. However, only a few reports have described prognostic models for hormone-naive progressive prostate cancer before treatment [10, 18–20].

Hussain et al. [21] reported various risk predictors of OS (SWOG9346). They identified baseline variables, such as bone pain, performance status, Gleason sum, weight change, positive lymph node metastasis, pre-study PSA increments, and PSA levels after treatment as strong prognostic factors for OS. Coopeberg et al. [9] also reported a large study on prostate cancer prognosis in hormone-naive patients in Japan and the USA. They assessed 13,740 US men and 19,265 Japanese men with prostate cancer and developed the Japan Cancer of the Prostate Risk Assessment (J-CAPRA). The CAPRA score, which ranges from 0–12, and is based on the Gleason sum, serum PSA levels at initial treatment, and clinical stage, can predict progression-free survival after primary androgen deprivation therapy. Although the endpoint of the J-CAPRA is progression-free survival, we are interested in developing an OS prognostic model for patients with hormone-naive metastatic prostate cancer. Progression-free survival is predictive of OS in men with CRPC [22], although the association between progression-free survival and OS is relatively weak. Some reports have indicated improvement in OS without an increase in progression-free survival [23] or improvement in progression-free survival without an increase in survival [24]. Accurate prediction models for prostate cancer survival probability would be helpful for counseling of patients.

In previous studies, EOD was reported to be a strong predictor of survival [7, 8]. EOD classification is used for evaluation of bone metastases. Because EOD is a subjective and semi-quantitative factor, more accurate and objective parameters for evaluation of spread of bone metastasis are warranted. Recently, the BSI, using a computer-aided diagnosis system for bone scans, has been expected to be an objective and quantitative clinical tool for evaluation of bone metastatic prostate cancer. The BSI is useful for prediction of survival in men with prostate cancer in various situations, such as hormone-naive cancer or CRPC [11, 13, 14, 25–29]. In this study, we measured BSI using BONENAVI version 2, which is a computer-assisted diagnosis system for bone scanning. BONENAVI version 2 software is based on a multi-center database and it showed excellent sensitivity and specificity for diagnosis of skeletal metastasis [17, 30].

In the hormone-naive setting, usefulness of the BSI as prognostic marker was reported by Koboteh and Poulsen [13, 27]. Koboteh and colleagues [27] reported that the BSI and Gleason scores were independent predictive factors of survival in men with a high risk of prostate cancer who were treated by androgen ablation therapy, although clinical stage and pre-treatment PSA levels were not prognostic factors. Poulsen et al. [13] also reported that only the BSI was an independent prognostic factor for survival of men with metastatic prostate cancer, although PSA and Gleason scores were not.

In our study, we analyzed the correlations between survival and clinical factors, including age, pre-treatment PSA levels, Gleason scores, clinical TNM classifications, and the BSI using the Cox proportional hazard regression model. EOD scores were significantly associated with the BSI value. EOD scores were not included in multivariate analysis because of multicollinearity. In results, patients’ age and the BSI were independent prognostic factor for OS of men with metastatic, hormone-naive prostate cancer. Because the BSI is continuous variables, the BSI is more ideal and accurate for prediction of individual patient’s prognosis compared with EOD scores. Moreover, we evaluated the discriminatory ability of our models by quantifying the C-index. The C-index of our model including BSI was 0.801 for OS. We also analyzed the discriminatory ability of the model excluding BSI and the model including EOD instead of BSI by quantifying the C-index. The C-index of the model excluding BSI was 0.751 for OS. The C-index of the models including EOD instead of BSI was 0.811.

A limitation of this study is the fact that patients who were enrolled in the study had various health statuses and complications. Our models did not consider health status or patients’ complications that may affect prostate cancer treatment outcomes [21, 31, 32]. Patients with prostate cancer are much older than those with other malignancies. Health status and complications should be classified in the rating score and included as predictive factors in the prognostic model. Our study did not include blood data. In previous studies, blood data, such as serum hemoglobin, alkaline phosphatase, lactate dehydrogenase, and C-reactive protein were reported as survival predictive factors for patients with prostate cancer [19, 20, 33]. Bone pain at diagnosis is also a strong predictor of survival [21]. Unfortunately, data regarding pain at baseline were not available in this study. Lack of data about PSA kinetics was also limitation of our study. Finally, our population was small, observation periods were relatively short, and number of event was also small. Thus, some of the estimates have wide confidential intervals. More large populations and long observations are required for establishing the usefulness of the BSI as a prognostic factor. Although some study limitations exist, the BSI before treatment might be useful for a prognostic biomarker of hormone-naive, bone metastatic prostate cancer.

## Conclusion

The pretreatment BSI and patients’ age are independent prognostic factors for patients with hormone-naive, bone metastatic prostate cancer. Compared with EOD, the BSI is a quantitative and objective factor for assessing bone metastases, and a more ideal factor for predicting prognosis in men with metastatic, hormone-naive prostate cancer.

## Abbreviations

BSI: bone scan index
CI: confidential interval
C-index: concordance index
CRPC: castration-resistant prostate cancer
EOD: extent of disease
HR: hazard ratio
J-CAPRA: Japan Cancer of the Prostate Risk Assessment
OS: overall survival
PSA: prostate-specific antigen
PSA: prostate-specific antigen
